# Efficiency of Airborne Sample Analysis Platform (ASAP) bioaerosol sampler for pathogen detection

**DOI:** 10.3389/fmicb.2015.00512

**Published:** 2015-05-27

**Authors:** Anurag Sharma, Elizabeth Clark, James D. McGlothlin, Suresh K. Mittal

**Affiliations:** ^1^Department of Comparative Pathobiology, College of Veterinary Medicine, Purdue University, West LafayetteIN, USA; ^2^School of Health Sciences, College of Health and Human Sciences, Regenstrief Center for Healthcare Engineering, Purdue University, West LafayetteIN, USA

**Keywords:** bioterrorism, airborne pathogens, air samples, ambient air sampler, virus detection, pathogen detection, pandemic, bioaerosol

## Abstract

The threat of bioterrorism and pandemics has highlighted the urgency for rapid and reliable bioaerosol detection in different environments. Safeguarding against such threats requires continuous sampling of the ambient air for pathogen detection. In this study we investigated the efficacy of the Airborne Sample Analysis Platform (ASAP) 2800 bioaerosol sampler to collect representative samples of air and identify specific viruses suspended as bioaerosols. To test this concept, we aerosolized an innocuous replication-defective bovine adenovirus serotype 3 (BAdV3) in a controlled laboratory environment. The ASAP efficiently trapped the surrogate virus at 5 × 10^3^ plaque-forming units (p.f.u.) [2 × 10^5^ genome copy equivalent] concentrations or more resulting in the successful detection of the virus using quantitative PCR. These results support the further development of ASAP for bioaerosol pathogen detection.

## Introduction

In the current global geopolitical environment, the threat of a terrorist attack on the US and international community has never been greater. A biological threat of great concern to public safety is the use of an aerosolized pathogenic micro-organism that can be spread in the air as happened with the anthrax attack of 2001 (Zink, 2011; Menrath et al., 2014). In addition, an individual infected with a deadly pathogen can transmit the pathogen at a high human density place or event. Some highly contagious and fatal biological agents that are considered as bio-terror agents include *Bacillus anthracis, Yersinia pestis*, small pox, or Marburg viruses and other emerging pathogens (Pohanka and Kuca, 2010; Menrath et al., 2014).

Besides the intentional release of bioaerosols, natural outbreaks of infectious diseases may occur without warning. Outbreaks including Severe Acute Respiratory Syndrome (SARS) in 2002–2004 (Christian et al., 2004), continuously evolving influenza virus strains (Khanna et al., 2008) and recent infections with Middle East respiratory syndrome coronavirus (MERS-CoV; Hartl, 2013) underscore that such a natural threat is a real possibility. Early detection of the pathogen can result in prompt intervention and treatment of exposed individuals as well as confine the secondary spread. Because of the high global mobility of people, the failure to detect an intentional or natural outbreak of a contagious disease could result in its rapid spread with catastrophic consequences. In order to effectively safeguard against such threats, it is critical to continuously monitor the air for the presence of pathogenic biological agents, particularly at high risk events or places such as airports, malls, subway stations, cruise ships, aircraft, theaters, stadiums, or schools. Such an approach requires an advanced system that can continuously sample the ambient air for pathogen detection.

Following the acquisition of an air sample, many different approaches including genome detection (Pyankov et al., 2007; Goransson et al., 2012; Usachev and Agranovski, 2012), immune (McBride et al., 2003; Rider et al., 2003; Skottrup et al., 2008), optical (Pan et al., 2003; Sengupta et al., 2007), or other biochemical assays (Hagleitner et al., 2001; Ho, 2002) have been employed for specific detection of a large selection of pathogens including bacteria, fungi, virus, or toxins. Since nucleic acids are the absolute biomarkers, quantitative polymerase chain reaction (qPCR) or quantitative reverse transcriptase-PCR (qRT-PCR) is strongly established as the method of choice for accurate, sensitive and specific identification of a number of pathogens in the same sample (Oppliger et al., 2011).

**FIGURE 1:**
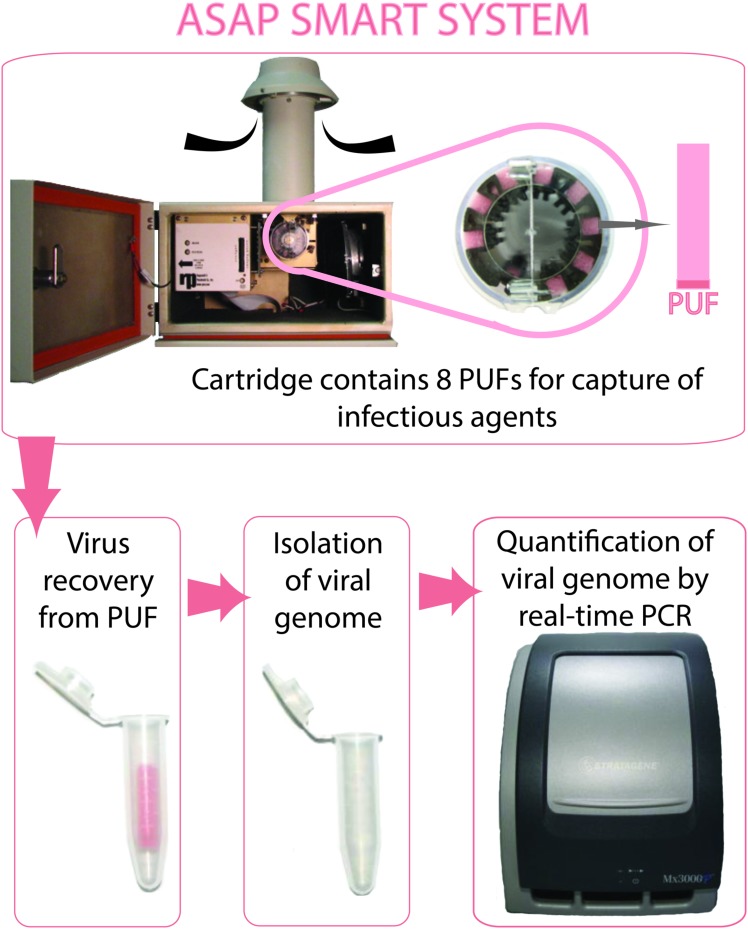
**Functioning of Airborne Sample Analysis Platform (ASAP) System.** Bioaerosols contained in the ambient air are sucked in through the inlet of the ASAP bio-sampler and deposited on a strip of polyurethane foam (PUF) contained in the Integrated Bio Aerosol Sampling System (iBASS) cartridge. The individual polyurethane foam (PUF) strips are then processed for pathogen isolation and nucleic acid extraction. Quantitative polymerase chain reaction (qPCR) is set up for specific detection of the pathogen.

The Airborne Sample Analysis Platform (ASAP) 2800 (Thermo Scientific, USA) bioaerosol sampler provides an effective means of collecting representative samples of air for subsequent laboratory analysis. At a flow rate of 200 l/min, this sampler pulls in the air which impacts on a piece of polyurethane foam (PUF) inside a cassette called Integrated Bio Aerosol Sampling System (iBASS) cartridge (**Figure 1**). In this study we investigated the efficiency of the ASAP system to acquire an aerosolized viral pathogen and detect it using a Taqman qPCR. For testing, we aerosolized an innocuous replication-defective bovine adenovirus serotype 3 (BAdV3) in a controlled laboratory environment. The ASAP system successfully trapped the virus-loaded aerosols which were subsequently detected by qPCR.

## Materials and Methods

### Surrogate Virus and Cell Line

Replication-defective BAdV3 with E1 and E3 deletions (BAd-ΔE1E3) was constructed and propagated in fetal bovine retinal cells transformed with adenoviral E1 (FBRT-HE1) as described elsewhere (van Olphen et al., 2002; Sharma et al., 2009). The FBRT-HE1 cells were grown as monolayer cultures in Eagle’s Minimum Essential Medium (MEM; Life Technologies, Gaithersburg, MD, USA) and supplemented with 10% reconstituted bovine serum (Fetal Clone III; Hyclone, Logan, UT) and 50 μg/mL gentamycin. The virus purification was done by cesium chloride-density gradient centrifugation as previously described (Graham and Prevec, 1991). The titration of virus was done in FBRT-HE1 cell lines by plaque assay and expressed as plaque forming units (p.f.u.)/mL. The DNA from the purified virus was isolated, and genome copy equivalent of the virus was calculated with the following formula where the ratio of genome copy number to p.f.u was 40.

$$Number\,\;\;of\,\;virus\,\;genome\,\;copies=\frac{\left(DNA\,\;amount\,\;(ng)\;\times 6.022\times 10^{23}\right)}{\left(Lenght\,\;(bp)\times 10^{9}\times 650\right)}$$

### Isolation of Viral DNA

Purified BAd-ΔE1E3 particles at various dilutions (0, 5 × 10^1^, 5 × 10^3^, 5 × 10^5^, or 5 × 10^7^ p.f.u.) in 50 μL were spiked in the strips of PUF of the ASAP system. Virus was recovered by adding 500 μl PBS to the PUFs and squeezing the liquid out of the PUFs by centrifugation. Un-spiked virus samples served as controls for the determination of virus recovery from the PUFs. DNA was isolated using the DNAzol reagent (Molecular Research Center). The isolated DNA was resuspended in 25 μL of distilled water, and the amounts of extracted nucleic acid were quantified by a qPCR assay using known amounts of purified BAd-ΔE1E3 nucleic acid as standards.

The efficiency of virus recovery was calculated as –

$$Efficiency=\frac{Copy\,\;number\,\;of\,\;virus\,\;genome\,\;re\,\mathrm{cov}ered\,\;from\,\;spiked\,\;samples}{Copy\,\;number\,\;of\,\;virus\,\;genome\,\;re\,\mathrm{cov}ered\,\;from\,\;purified\,\;virus}\times 100$$

### Primers and Taqman Probes

Specific primers and a Taqman probe targeting the E4 region of BAdV3 were designed using Primer Express 2.0 software (Life Technologies). The sequence of primers and probes are as follows:

Forward primer – 5′- GGGCGAGCAATCAGCTCTTA – 3′

Reverse primer – 5′- CTAATCCACTGCCCATGTACACA – 3′

Probe – 5′- AGTCCCTGCCCACTTTTGCCTGG – 3′

The oligonucleotides were synthesized by Applied Biosystems. The probe was labeled with FAM (6-carboxyfluorescein) at the 5′ end and with MGB (minor groove binder) at the 3′ end. The primers and probes were reconstituted in TE buffer, aliquoted, and stored at -20°C until required.

### Quantitative PCR

For absolute quantification of the viral genome, a standard curve was obtained using serial 10-fold dilutions (three copies to 3 × 10^7^ copies in 10 μl) of purified genomic DNA of BAd-ΔE1E3. The copy number of the viral genome was calculated based on spectrophotometric quantification and the molecular mass of BAd-ΔE1E3’s genomic DNA. A standard curve was run for each set of assays. For qPCR, 10 μL of isolated DNA was used in a 25 μL reaction using Taqman PCR core reagents (Applied Biosystems, Foster City, CA, USA). The reaction mixture contained 10x Taqman buffer, 250 nM of forward and reverse primers, and 100 nM of Taqman probe along with other standard kit components. Each reaction was carried out in duplicate. The qPCR was performed using the Mx3000 Thermocycler (Stratagene, Cedar Creek, TX, USA) with the following reaction conditions: 50°C for 2 min, followed by polymerase activation (95°C for 10 min), 45 cycles of denaturation (95°C for 15 s) and annealing/extension (60°C for 1 min). The threshold cycle (Ct) value for individual reactions was determined, and data was analyzed with MxPro software to obtain the absolute copy number of viral genome.

### Evaluation of the Efficiency of Airborne Sample Analysis Platform (ASAP System) in Trapping Aerosolized Virus Particles

The basic procedure used to test the efficiency of the ASAP System is depicted in **Figure 1**. The ASAP System was placed in a biosafety cabinet [72 inches (183 cm) × 24 inches (61 cm) × 28 inches (71 cm)] and various dilutions [0, 5 × 10^1^, 5 × 10^2^, 5 × 10^3^, 5 × 10^4^, 5 × 10^5^, 5 × 10^6^, or 5 × 10^7^ p.f.u. (i.e., 0, 2 × 10^3^, 2 × 10^4^, 2 × 10^5^, 2 × 10^6^, 2 × 10^7^, 2 × 10^8^, or 2 × 10^9^ genome copies equivalent)] of BAd-ΔE1E3 in 0.5 ml were aerosolized with the help of a vibrating mesh nebulizer system (Micro Air, Omron) which was kept at distances of six inches (15 cm), one foot (30 cm), and 4 feet (122 cm) from the air intake trap of the machine. The machine was allowed to run for an additional 15 min after the completion of the aerosolization of each sample. Each dilution was run in triplicate. One PUF per cartridge was not exposed to the aerosols and was kept as a negative control. After the run, each PUF was collected from the iBASS cartridge. The nucleic acid was extracted from the virus trapped in the PUF using the DNAzol reagent as per the manufacturers’ protocol and resuspended in 25 μL of distilled water. The amounts of recovered nucleic acid were quantified by qPCR.

In addition, to mimic the conditions in a normal work environment, the same experiment was also conducted in a room [9 feet (274 cm) × 10 feet (305 cm) × 10.8 feet (329 cm)] with a regular ventilation system. This time the aerosols were created by placing the nebulizer at greater distances [5 feet (152 cm) and 10 feet (305 cm)] from the ASAP system. ASAP was allowed to run for an additional 1 h after completion of the aerosolization of each sample. This was followed by extraction of nucleic acid and quantitation by qPCR. All experiments including aerosolization of virus were conducted as per approved institutional biosafety protocols.

## Results

### Selection of a Surrogate Virus

**FIGURE 2:**
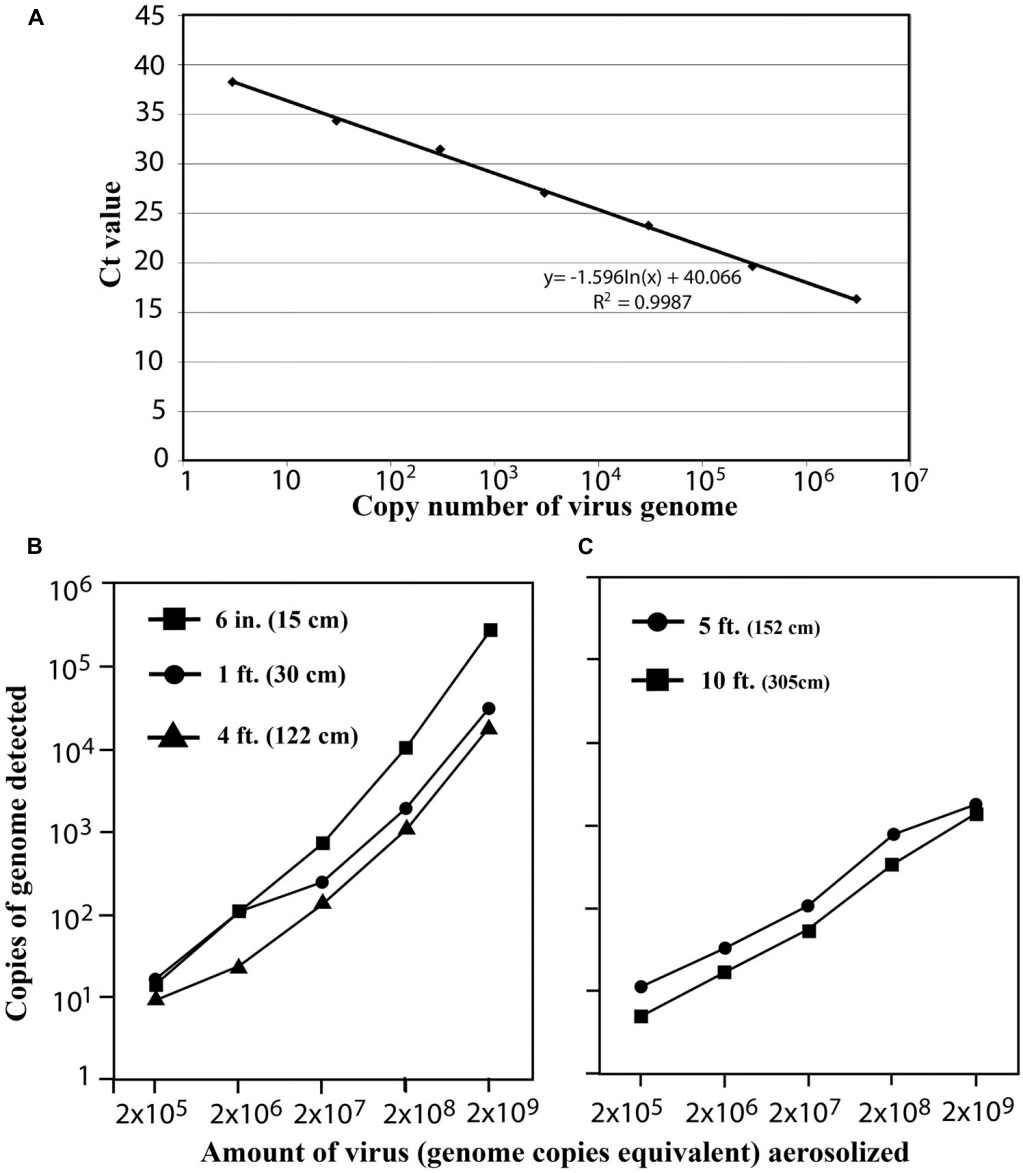
**(A)** Standard curve for absolute quantification of BAd-ΔE1E3 genome. Tenfold serial dilutions of BAdΔE1E3 genomes were subjected to Taqman qPCR, and the Ct values were plotted against the absolute numbers of viral genome. **(B,C)** Detection efficiency of aerosolized BAd-ΔE1E3 virus by ASAP System at different distances. Different concentrations of BAd-ΔE1E3 virus were aerosolized at different distances from the air inlet of the ASAP System, placed in **(B)** biosafety cabinet [72 inches (183 cm) × 24 inches (61 cm) × 28 inches (71 cm)], or **(C)** in a room [9 feet (274 cm) × 10 feet (305 cm) × 10.8 feet (329 cm)]. The amount of BAd-ΔE1E3 virus trapped on the PUFs was quantified by qPCR and depicted as geometric means.

In order to explore the usefulness of the ASAP system as a rapid detection system for highly pathogenic agents in air samples, we needed a surrogate virus which was safe but could mimic the air sampling procedure that will be required for detecting highly pathogenic infectious agents. We selected a replication-defective bovine adenovirus (BAd-ΔE1E3) having deletions in the early region (E) 1 (E1) and E3. The E1 gene products are essential for adenovirus replication; therefore, such E1-deleted viruses can be grown only in cell lines that constitutively express E1. Even wild type BAdV3 is not pathogenic in human or animals. For quantification of BAd-ΔE1E3 genomes by qPCR, a standard curve was generated using serial dilutions of known amounts of BAd-ΔE1E3 genomes as standards (**Figure 2A**). The detection limit of our qPCR assay was three genomes of BAd-ΔE1E3.

### Nucleic Acid Recovery from PUFs of ASAP System

Since the sensitivity of the ASAP system also depends on the efficiency of nucleic acid recovery from the PUFs containing the trapped infectious agent, we spiked various amounts of purified BAd-ΔE1E3 onto the PUFs and processed them for nucleic acid extraction by DNAzol (Molecular Research Center). The amounts of extracted nucleic acid were quantitated by qPCR, and known amounts of purified BAd-ΔE1E3 nucleic acid served as standards. The recovery of nucleic acid by this method was approximately 88% (**Table 1**).

**Table 1:** Efficiency of recovery of BAd-ΔE1E3 virus from PUF.

Virus amount (p.f.u./50 μl)	Copy number of virus genome recovered		Percent recovery
	Pure virus	Spiked	
5.0E+01	3.01E+01	2.58E+01	85.54
5.0E+03	1.09E+02	1.09E+02	99.81
5.0E+05	3.93E+04	2.95E+04	75.21
5.0E+07	6.50E+06	5.94E+06	91.28

### Evaluation of the Efficiency of ASAP System in Trapping Aerosolized Virus Particles

Before conducting experiments, we determined the efficiency of the ASAP system in trapping aerosolized virus particles in addition to the likelihood of cross-contamination from one PUF to another PUF in the iBASS cartridge. The ASAP System was placed in a biosafety cabinet and various amounts (0, 2 × 10^3^, 2 × 10^4^, 2 × 10^5^, 2 × 10^6^, 2 × 10^7^, 2 × 10^8^, or 2 × 10^9^ genome copies equivalent per 0.5 ml) of BAd-ΔE1E3 were aerosolized with the help of a nebulizer kept at distances of six inches (15 cm), 1 foot (30 cm), and 4 feet (122 cm) from the air intake trap. Each sample was run in triplicate. After the run, each PUF was collected from the cartridge, and the nucleic acid was extracted from the virus trapped in the PUF using the DNAzol protocol. The amounts of the recovered nucleic acid were quantitated by qPCR assay.

The ASAP biosampler efficiently collected and trapped viral aerosols from the air. The minimum of 2 × 10^5^ genome copies equivalent (5 × 10^3^ p.f.u.) of the input virus was detected (**Figure 2B**). The amount of viral genome detected decreased proportionately with the distance from the ASAP air inlet. However, even at increased distance, the ASAP system was sensitive enough to detect as low as 2 × 10^5^ genome copies equivalent (5 × 10^3^ p.f.u.) of virus input. There was no cross-contamination from one PUF to another; the negative PUF in a cartridge remained negative after each run.

Subsequently, to mimic conditions similar to a normal work environment, the experiment was conducted in a room with a normal ventilation system. The ASAP system was placed in the room, and increasing amounts of BAd-ΔE1E3 were aerosolized with the help of a nebulizer kept at distances of 5 feet (152 cm) and 10 feet (305 cm) from the machine. Similar to the experiment in the biosafety cabinet, the ASAP sampler efficiently collected and trapped the viral aerosols from the air. In each experiment, there was a dose-dependent increase and a distance-dependent decrease in the number of genomes detected (**Figure 2C**). The minimum detection limit remained unchanged at 2 × 10^5^ genome copies equivalent (5 × 10^3^ p.f.u.) These results show that the ASAP bioaerosol sampler can efficiently monitor ambient air for viral pathogens.

## Discussion

Over the last several decades, continuous air sampling coupled with rapid and accurate detection of potential pathogens in bioaerosols has attracted much attention. The ASAP bioaerosol sampler has been tested to sample bacteria and spores (Goransson et al., 2012) or metalliferous airborne particles (Moreno et al., 2015) under field conditions. However, the ASAP sampler had not yet been tested for collection of viral aerosols. In this study we investigated the efficacy of the ASAP bioaerosol sampler to collect and trap surrogate virus aerosols from the air and detect them using suitable assays. We demonstrated that in controlled laboratory conditions, the ASAP sampler can efficiently trap surrogate BAd-ΔE1E3 viral aerosols that can be extracted from the PUFs and identified using qPCR.

The purpose of bioaerosol sampling is to detect a biological agent release so that the impact of a biological agent attack can be reduced by executing appropriate responses with minimal delay. However, the conventional time-consuming laboratory methods involving culturing of microorganisms can take days to identify the pathogen. Molecular biology techniques such as qPCR are rapid, specific and sensitive enough to identify the genus, species or strain of the pathogen. These molecular assays can also be multiplexed to monitor more than one bio-threat agent at a time. As qPCR assays are quantitative, they can provide additional valuable information regarding the concentration of pathogens in the ambient air. We successfully detected the bioaerosols containing as low as 2 × 10^5^ genome copies equivalent (5 × 10^3^ p.f.u.) of BAd-ΔE1E3. The technology of airborne pathogen detection is still in the trial stage with no approved device in practical application yet. Unfortunately, there are no set standards for the sensitivity of airborne pathogen detection. However, the detection of surrogate virus, released from a distance of 10 ft (305 cm), and at a concentration as low as 2 × 10^5^ genome copies equivalent (5 × 10^3^ p.f.u.) in a room the size of 972 cubic ft (27.5 cubic meters) is quite sensitive by many standards.

The majority of the currently available air sampling platforms are based on either impaction onto agar, impingement into liquid, or dry impaction (Kesavan and Sagripanti, 2015), and each has its limitations. The direct collection of microbes on agar is limited since not all species can be cultured and others have distinct nutrient requirements. The agar surface can also get saturated in areas with high concentrations of microbes, and it cannot be used for viruses.

For viruses and other viable microorganisms, the collection of aerosols in liquid media/buffer is a preferred method. However, the high rate of sample collection usually results in violent bubbling of the collection fluid resulting in considerable loss of collection fluid within short time (Agranovski et al., 2004). Therefore, samplers based on liquid impinging or bubbling usually operate at lower speeds. Some advancement has been made to address these problems.

In the dry method (this study), the high volume of air impacting on the PUF results in a strong desiccation effect during sampling which dramatically diminishes the viability of most microorganisms. The inefficiency of dry impaction to collect viable particles is often considered a limitation; however, this method allows high rates of sample collection (>200 l/min) enabling rapid analysis of a larger sample volume from the environment.

The advantage of molecular techniques is that the genetic material can be detected even from inactive microorganisms. The specific detection of the genome of potentially dangerous microorganisms in the aerosol samples is evidence of a direct threat serious enough to raise an alarm and initiate appropriate measures. In this study we use only a DNA virus to test the efficacy of ASAP sampler. The iBASS cartridge is removed after every 4–8 h of operation for analysis of the microorganisms collected on the PUF, and during this time the genetic material, even from the RNA viruses, is expected to remain stable inside the capsid/shell of the inactive virus. It has been shown that phage PR772 and possibly other viruses as well, lost their infectivity before the genome degradation (Turgeon et al., 2014). Additionally, it has been demonstrated that the overall recovery of airborne viruses should be estimated based on the detection of genetic material rather than on viral infectivity (Verreault et al., 2010; Turgeon et al., 2014). The use of molecular biological methods is more likely to avoid false negative results than infectivity assays for the detection of airborne viruses (Verreault et al., 2010).

Aerosols are impacted on PUF which is often considered superior to other substrates such as glass bead beds, cellulose filters and other solid substrates. In particular, the PUF has minimum particle bounce and re-entrainment losses and has high collection efficiency without the need for greasing or oiling the substrate (Crook et al., 1997; Kavouras and Koutrakis, 2001; Lee et al., 2005). Furthermore, there is no pressure drop increase with particle accumulation, and it is easy to recover the impacted particles from the PUF.

In contrast to the ASAP bioaerosol system described in this study, some samplers utilize a combination of immunoassay and PCR to increase reliability and minimize the possibility of false positives (Hindson et al., 2005). Furthermore, the number of agents that can be detected in a sample is limited by the number of primer/probe sets used for multiplexing. If the primer/probe for a specific pathogen is not included in the mix, it would not be detected. It is expected that in the future pathogen detection will be based primarily on next-generation sequencing (NGS; Frey et al., 2014; Lecuit and Eloit, 2014). A key advantage of NGS is that it allows identification of a range of organisms (bacteria, viruses, fungi). Subsequent adaptations to couple the ASAP bioaerosol sampler with NGS platforms can potentially further enhance its accuracy, sensitivity and spectrum of detection.

The ASAP sampler is highly portable (32 cm/side; 13.6 kg), quiet in operation and has low energy usage (battery operable). The ASAP system equipment is extremely user friendly and allows changing the default settings of the machine to add attachments such as a GPS, wireless and Ethernet communication, temperature, and wind sensor, video exposure monitoring with regular and infrared cameras to identify anyone with fever who may be a carrier of a pathogen, and real-time sensors for radioactivity. ASAP can be controlled using an external trigger. All of these aspects of the ASAP 2800 system would make it a dynamic and powerful tool in the detection of airborne pathogens. The operation of the sampler can be adjusted depending on the requirement of the facility, for example – 1 h for each PUF for a total 8 h run or half an hour per PUF for a 4 h run. Sample analysis can be done once every 4–8 h or more frequently depending on the need of the facility. Once released, the virus-loaded fine bioaerosol particles can remain suspended for hours (Yang et al., 2011) and available for detection by bioaerosol samplers. At the end of each run, the sealed iBASS cartridge can be aseptically removed and immediately analyzed for the presence of any alarming genetic material. The rotating iBASS cartridge can hold as many as eight different sampling PUFs along with a blank and negative control. The option of eight different samples allows the selection of specific times to acquire samples. Since there is no cross contamination between the PUFs in an iBASS cartridge, the sampling at different times can be useful to detect the time period when the airborne pathogen was brought into the area. Standard operating procedures for an ASAP system have already been developed by Lovelace Respiratory Research Institute.

For maximum efficacy in larger environments such as airports or stadiums, the bioaerosol samplers can be installed at strategic locations and work in conjunction with additional security surveillance such as video, personnel, and canine units. The ASAP bioaerosol sampler can also be used for evaluation of microbial contamination in the working environments in agricultural (Popescu et al., 2014; Sondergaard et al., 2014) or industry (Skora et al., 2014) settings, investigation of microbial metagenomics at distinct environments (Be et al., 2015) or in hospitals to track, identify, and control nosocomial infections (Sudharsanam et al., 2012).

## Conclusion

Overall, these results suggest that the ASAP bioaerosol sampler is a promising system for monitoring ambient air that has been proven to work with viral pathogens. Additional testing to evaluate its feasibility in real world conditions such as small airports, hospitals, and military installations is recommended for its further development and subsequent use for the detection of real pathogens. This study also presents an ideal surrogate virus that can potentially be used in additional studies investigating viral aerosol sampling and detection. BAd-ΔE1E3 is a DNA virus that is quite stable in the environment. BAdV3 does not cause any harm to humans and to ensure additional safety it has been genetically modified to make it replication defective.

## Conflict of Interest Statement

The authors declare that the research was conducted in the absence of any commercial or financial relationships that could be construed as a potential conflict of interest.
